# Dynamic Transitions Between Brain States Predict Auditory Attentional Fluctuations

**DOI:** 10.3389/fnins.2022.816735

**Published:** 2022-03-18

**Authors:** Hirohito M. Kondo, Hiroki Terashima, Takahiro Ezaki, Takanori Kochiyama, Ken Kihara, Jun I. Kawahara

**Affiliations:** ^1^School of Psychology, Chukyo University, Nagoya, Japan; ^2^NTT Communication Science Laboratories, Nippon Telegraph and Telephone Corporation, Atsugi, Japan; ^3^Research Center for Advanced Science and Technology, The University of Tokyo, Tokyo, Japan; ^4^Brain Activity Imaging Center, ATR-Promotions, Seika, Japan; ^5^Department of Information Technology and Human Factors, National Institute of Advanced Industrial Science and Technology (AIST), Tsukuba, Japan; ^6^Department of Psychology, Hokkaido University, Sapporo, Japan

**Keywords:** brain dynamics, hearing, sustained attention, attentional fluctuation, gradual-onset continuous performance task, reaction time (RT), energy landscape analysis, functional magnetic resonance imaging (fMRI)

## Abstract

Achievement of task performance is required to maintain a constant level of attention. Attentional level fluctuates over the course of daily activities. However, brain dynamics leading to attentional fluctuation are still unknown. We investigated the underlying mechanisms of sustained attention using functional magnetic resonance imaging (fMRI). Participants were scanned with fMRI while performing an auditory, gradual-onset, continuous performance task (gradCPT). In this task, narrations gradually changed from one to the next. Participants pressed a button for frequent Go trials (i.e., male voices) as quickly as possible and withheld responses to infrequent No-go trials (i.e., female voices). Event-related analysis revealed that frontal and temporal areas, including the auditory cortex, were activated during successful and unsuccessful inhibition of predominant responses. Reaction-time (RT) variability throughout the auditory gradCPT was positively correlated with signal changes in regions of the dorsal attention network: superior frontal gyrus and superior parietal lobule. Energy landscape analysis showed that task-related activations could be clustered into different attractors: regions of the dorsal attention network and default mode network. The number of alternations between RT-stable and erratic periods increased with an increase in transitions between attractors in the brain. Therefore, we conclude that dynamic transitions between brain states are closely linked to auditory attentional fluctuations.

## Introduction

Sustained attention is essential to adaptive behaviors such as driving or listening to a lecture. Focused states of attention are often disturbed by external events and internal factors. Reduction of one’s attention level leads to “mind wandering,” cognitive errors, and even serious accidents (Edkins and Pollock, 1997; Cheyne et al., 2006; Smallwood and Schooler, 2015). From the perspective of vigilance decrements, human error research has investigated mistakes that occur only rarely over long periods of time (Mackworth, 1948; Esterman and Rothlein, 2019). However, this classical method is not sensitive to momentary changes in attentional states. One sophisticated study used a gradual-onset continuous performance task (gradCPT) to demonstrate moment-to-moment fluctuations of sustained attention (Esterman et al., 2013).

The gradCPT is an experimental paradigm that was designed for assessing temporal dynamics of sustained attention. Stimuli, such as visual images, in this task are presented continuously, overlapping one another. Participants quickly respond to frequent Go trials and withhold responses to infrequent No-go trials. Importantly, the false alarm (FA) rate of responses is closely linked to variability of reaction times (RTs) (Esterman et al., 2013). Previous studies have also found neural correlates of vigilant and sustained attention. The core network related to vigilant attention includes the frontal, parietal, and subcortical areas (Langner and Eickhoff, 2013). For the gradCPT, the dorsal attention network (DAN) (Corbetta and Shulman, 2002, 2011), including the frontal eye field and intraparietal sulcus, was activated during erratic responses, whereas the default mode network (DMN) (Fox et al., 2005), including the medial prefrontal cortex (mPFC) and precuneus (PCu), was activated during stable responses (Esterman et al., 2013; Fortenbaugh et al., 2018). In contrast, another study using multi-voxel pattern analysis revealed that activations in the DAN and DMN did not distinguish attentional periods (Rosenberg et al., 2015). Thus, findings of previous studies using visual gradCPTs have been mixed, and different approaches are needed to gain new insights into the properties of sustained attention.

This functional magnetic resonance imaging (fMRI) study focused on temporal dynamics of brain activity, particularly in an auditory domain. We used an auditory gradCPT, the performance of which was correlated with visual gradCPT performance (Terashima et al., 2021). Participants indicated using a button press whether a voice was male or female for each trial, while listening to a sequence of narrations. For each participant, we obtained time-series data of RT variability from the auditory gradCPT. Intra-individual variability reflects the efficiency of attentional resources assigned to cognitive demands (Stuss et al., 2003; Klein et al., 2006). The fluctuations of RTs occurred between a stable period and an erratic period, which are termed “in the zone” and “out of the zone” periods (Esterman et al., 2013). We computed the variance time course (VTC) of RTs and specified VTC-related brain regions that are relevant to maintenance of attentional levels.

We further checked whether dynamic features of brain activations explained auditory gradCPT performance: FA rate, correct rejection (CR) rate, sensitivity (*d*′), and the number of alternations between in- and out-of-the-zone periods. To probe an interaction between the DAN and DMN, the following brain areas were chosen as regions of interest (ROIs): the superior frontal gyrus (SFG) including the frontal eye field, superior parietal lobule (SPL) including the intraparietal sulcus, mPFC, PCu, and superior temporal gyrus (STG). A previous study using energy landscape analysis showed that dynamic transitions of frontal-area and visual-area states could predict individual differences in bistable visual perception (Watanabe et al., 2014). This indicates that perceptual organization is related to underlying brain dynamics. Thus, it is reasonable to assume such a link between task performance and brain dynamics. Brain dynamics are displayed as a series of stays and transitions between different attractors in the energy landscape (Watanabe et al., 2014; Watanabe and Rees, 2017; Ezaki et al., 2018). We examined how dynamic transitions between task-related activities contribute to auditory gradCPT performance.

## Materials and Methods

### Participants

Twenty-nine participants (15 males and 14 females; mean ± SD age = 25.5 ± 4.4 years, range 20–35 years) were recruited for the present study. They were right-handed, healthy Japanese people with normal hearing. According to *a priori* power analyses with a power of 0.8 (α-level = 0.05), we required at least 29 participants to detect significant correlations (*r* = 0.5; bivariate normal model). The present study was approved by the Research Ethics and Safety Committees of Chukyo University and ATR-Promotions (approval nos. RS20-017 and AN21-056). Experimental procedures were implemented in accordance with Ethical Guidelines for Medical and Biological Research Involving Human Subjects. All participants gave written informed consent after the procedures were fully explained to them.

### Stimuli and Procedures

**FIGURE 1:**
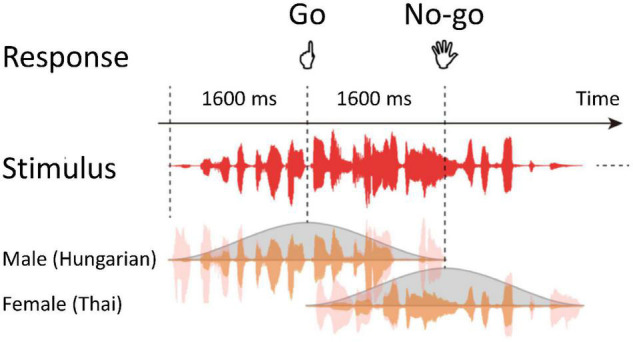
Schematic representation of the auditory gradual-onset, continuous performance task (gradCPT). Stimuli temporally overlapped. Participants judged the genders of voices for each trial.

Stimuli of the auditory gradCPT consisted of sequential narrations by a male (90%) and female (10%) that gradually changed from one to the next (Terashima et al., 2021). Narrations of ten males and ten females chosen from a language database, excluding Japanese narrations, were randomly presented through a run (Figure 1). Thus, using phonetic features of stimuli, for each trial, participants judged whether a voice was male or female. The sound pressure level of all narrations was adjusted to a comfortable listening level. Stimuli were delivered through plastic tubes and headphones (Hitachi Advanced Systems, Yokohama, Japan). The same stimuli were not repeated in succession.

Participants were instructed to press a button as quickly and accurately as possible if they heard male narrations (Go trials) and to refrain from responding for female narrations (No-go trials). They responded with their left index fingers to avoid affecting the activity of language areas in the left hemisphere. A response deadline was implicit in the task because the current stimulus was replaced by the next stimulus within 1.6 s of stimulus onset asynchrony.

We obtained blood-oxygen-level-dependent (BOLD) signals during auditory gradCPT. An fMRI session comprised two experimental 410-s runs, including 250 trials for each. The duration of a single stimulus was 3.2 s. The order of the stimuli was randomized across runs. Practice trials preceded the experimental runs to familiarize participants with the task. We managed stimulus presentation and response collection using Presentation software (Neurobehavioral Systems, Berkeley, CA, United States).

### Imaging Data Acquisition

We scanned participants during the task using a MAGNETOM Prisma (Siemens, Munich, Germany), a 3T MRI scanner with a body coil as a transmitter and a 20-channel head coil as a receiver. We placed small, comfortable, elastic pads on both sides of participants’ heads to minimize head motion. For an assessment of cortical thickness and volume, we acquired three-dimensional anatomical images of the whole brain with a T1-weighted MPRAGE sequence: repetition time (TR) = 2,250 ms; echo time (TE) = 3.06 ms; inversion time = 900 ms; flip angle = 9 deg; 208 sagittal slices; matrix size = 256 mm × 256 mm; isotropic voxel size = 1 mm^3^.

For each run, we acquired 205 volumes using the multi-band, echo-planar imaging (EPI) sequence. Functional images sensitive to the BOLD response covered the whole brain: 72 consecutive slices parallel to the plane of the anterior-posterior commissure. A T2*-weighted EPI sequence was used with the following parameters: TR/TE = 2000/30 ms; flip angle = 80 deg; multiband acceleration factor = 3; partial Fourier = 6/8; matrix size = 100 × 100; voxel size = 2 mm × 2 mm × 2 mm. At the beginning of an fMRI session, we acquired a B0 field map to correct for geometric distortions: TR/TE1/TE2 = 750/5.17/7.63 ms; flip angle = 50 deg; matrix size = 100 × 100; 72 slices in the same orientation and geometry as the EPI sequence.

### Behavioral Data Analysis

We defined an RT as the relative time from stimulus onset to button press (Esterman et al., 2013; Terashima et al., 2021). The time window to assign the response to each stimulus was set from 70% of the appearance phase to 40% of the disappearing phase. That is, we assigned all button presses in the time window to the corresponding trial. We used the shortest RT for a subsequent analysis when finding multiple button presses in a single time window. On the basis of the response assignment, we classified all trials into hit, miss, FA, or CR trials. For each participant, we calculated *d*′ and the median of RTs. We focused on trial-by-trial RT variability because we were also interested in the fluctuation of sustained attention within participants. We computed *z*-scored RTs in each run, transformed them into absolute values, and called the time-series data the VTC. Using a median split, we divided performance into low- and high-variability epochs, which were termed in- and out-of-the-zone periods, respectively. RTs that were too quick or too slow could be considered a signature of attentional fluctuation. We smoothed the VTCs with a Gaussian kernel at full width at half maximum of 7 s. Statistical analyses were carried out using IBM SPSS Statistics (ver. 25).

### Imaging Data Analysis

We analyzed fMRI data using SPM12^1^ and in-house codes implemented in MATLAB R2019a (MathWorks, Natick, MA, United States). For each run, we discarded the five initial images to achieve steady-state equilibrium between radio-frequency pulsing and relaxation. For preprocessing, we performed slice-timing correction, realigned all functional images to correct for head motion, and unwarped them to remove dynamic EPI distortions (movement-by-susceptibility interactions). Head-motion correction included 24 realignment parameters, white matter, and cerebrospinal fluid as nuisance covariates. For three participants, maximum values of head movements were greater than either 1.5-mm transformations or 1.5-deg rotations within each run. We excluded these data from subsequent analyses, leaving 26 participants. A value (mean ± SD) of framewise displacement was 0.090 ± 0.039. We used a B0 field map processed using the FieldMap toolbox of SPM12 (Andersson et al., 2001; Hutton et al., 2002). All functional images were normalized to Montreal Neurological Institute (MNI) space, resampled to a voxel size of 2 mm × 2 mm × 2 mm, and smoothed with an isotopic Gaussian kernel of 6 mm full width at half maximum.

Using a general linear model, we entered the four types of trials into a design matrix (Worsley and Friston, 1995). Each trial type was embedded as a stick function. Trial-related regressors were convolved with a canonical hemodynamic response function (HRF). A high-pass filter with a cut-off period of 128 s was used to remove an artifact of the low-frequency trend. We calculated serial autocorrelation from pooled active voxels with a maximum likelihood procedure. Autocorrelation was applied to whiten the data of the design matrix (Friston et al., 2002). We obtained FA- and CR-related statistical maps at the first level and performed random-effects analyses to identify brain activations at the second level.

We constructed another design matrix to examine changes in BOLD signals corresponding to the VTC. We estimated the amplitude-modulated, non-smoothed VTC that was convolved with a canonical HRF. The time-delayed VTC was downsampled to 0.5 Hz (i.e., TR = 2 s) and was used as the regressor of the design matrix. We conducted random-effects analyses using VTC-related statistical maps.

### Energy Landscape Analysis

On the basis of automated anatomical labeling (Tzourio-Mazoyer et al., 2002), we defined five ROIs as follows: SFG, SPL, mPFC, PCu, and STG. To reduce the number of signals for a robust energy landscape analysis, we averaged BOLD signals of ROIs in the right and left hemispheres and fed the signals into the energy landscape analysis. Following procedures used in previous studies (Ezaki et al., 2017, 2018), we first binarized each of the five signals and concatenated the data of all participants. Second, appearance probabilities of brain activity patterns were fitted using the pairwise maximum entropy model (Boltzmann distribution; see section “Pairwise Maximum Entropy Model”). Third, using “energy” values defined with the fitting function, we constructed an energy landscape representation of activity patterns (see section “Construction of Energy Landscape”). Fourth, on the basis of the energy landscape, we divided activity patterns into discrete states, each of which corresponds to a basin of an energy local minimum. Finally, using the list of activity patterns in each discrete state, we obtained a coarse-grained representation of the original time series.

#### Pairwise Maximum Entropy Model

For each session, participant, and ROI, we computed the average value of the BOLD signal, which was then used as a threshold to binarize the signal into −1 (inactive) or +1 (active). For each volume, the brain state of the five ROIs was represented by an activity pattern σ=(σ_1_,σ_2_,…,σ_5_), where σ_*i*_(*i* = 1,…, 5) denotes the activity of *i*th ROI (inactive: σ_*i*_ = −1, active: σ_*i*_ = 1). We computed the empirical appearance probability of each of the 2^5^(=32) activity patterns by counting the number of appearances. This probability distribution was fitted with the following pairwise maximum entropy model:


$$P\,\left(\sigma \right)=\frac{\exp\left(-E\,\left(\sigma \right)\right)}{\sum_{\sigma }\exp\left(-E\,\left(\sigma \right)\right)},$$

where $E\,\left(\sigma \right)=-\sum_{i=1}^{5}h_{i}\,\sigma_{i}-\sum_{i=1}^{5}\sum_{\begin{array}{c} j=1\\ j\neq i \end{array}}^{5}J_{i\,j}\,\sigma_{i}\,\sigma_{j}$ is a function to compute the energy value defined for each activity pattern. We tuned the parameters of the model (*h*_*i*_ and *J*_*ij*_) using the gradient ascent algorithm that maximizes the likelihood function (Ezaki et al., 2017). The accuracy of fitting (*r*_*D*_) was sufficiently high (0.997). We used these energy values in the following analyses.

#### Construction of Energy Landscape

Here, we show that two activity patterns (σ =)α and (σ =)β are neighbors if and only if they differ only at a single ROI, i.e., if the Hamming distance between these activity patterns is equal to 1. Thus, each of the 32 activity patterns had five neighboring activity patterns. The algorithm to compute the energy landscape was as follows. (i) We selected an activity pattern from a list of 32 possible activity patterns. (ii) We checked the energy value of the selected activity pattern and its neighboring activity patterns and moved to one of these patterns that had the minimum energy value. (iii) We repeated (ii) until the current activity pattern was selected as the next move, which meant that that pattern was a local minimum, having a smaller energy value than that of its neighbors. (iv) We recorded this sequence of moves. (v) These procedures from (i) to (iv) were performed for all initial activity patterns. The resultant paths defined sets of activity patterns that belong to a basin of energy local minimum activity patterns. In this fashion, a single energy local minimum was associated with each activity pattern. A set of activity patterns that were associated with an energy local minimum is termed the basin of the local minimum. Using this labeling, we mapped BOLD signals to the time series of the label of basins. It should be noted that transition rates of the brain state between basins are a common measure used in the literature (Watanabe et al., 2014; Watanabe and Rees, 2017; Ezaki et al., 2018).

## Results

### Behavioral Performance

Behavioral performance (mean ± SD) for the auditory gradCPT generally reached a satisfactory level: 74.1 ± 14.6% for hit rate, 23.8 ± 11.7% for FA rate, and 1.56 ± 0.65 for *d*′. Due to scanner noise, *d*′ observed in the present study was worse than that (2.85 ± 0.82) obtained from an experiment outside the scanner: *t* = 6.42, *p* < 0.001, Cohen’s *d* = 1.77. The number of alternations between in- and out-of-the-zone periods was 32.2 ± 3.4 throughout 800-s sessions. Correlations between the task performance and alternation numbers did not reach statistical significance: |*r*| < 0.36, *p* > 0.07. The pattern of the results is consistent with previous findings obtained in a laboratory environment (Terashima et al., 2021).

### Imaging Results

**FIGURE 2:**
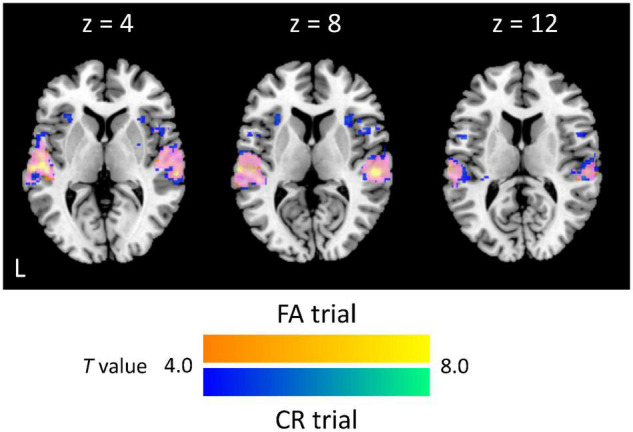
Extent and intensity of auditory-related activations. Activated areas during false alarm (FA) and correct rejection (CR) trials are colored yellow and blue, respectively, whereas overlapped activations are colored magenta (*p* < 0.05, corrected at the cluster level; *k* = 279 voxels).

We first specified trial-based brain activations. For both FA and CR trials, we found activations of the auditory cortex (Brodmann area: BA 42/41), STG (BA 22), right dorsolateral PFC (BA 46), supplementary motor area (BA 6), and premotor area (BA 6) (Table 1). Activated areas largely overlapped between FA and CR trials. Specifically, the activity of the auditory cortex was extended from the primary auditory area to the secondary auditory area, including the lateral area and posterior area (Figure 2). Local maxima of auditory-related activations were positioned at the lateral area, regardless of trial type, although additional activations were found in the anterior area of the auditory cortex during FA trials. Cognitive subtraction analyses revealed that significant activations did not survive, in contrast to FA vs. CR trials. Thus, for auditory gradCPT, unsuccessful inhibition of prepotent responses shares similar mental processes with successful inhibition of prepotent responses.

**TABLE 1:** Brain regions activated during false alarm and correct rejection trials.

Brain region	BA	*X*	*Y*	*Z*	*T*-value
**False alarm trials**					
Prefrontal cortex	R46	38	50	24	4.65
Premotor area	R6	54	10	44	5.38
Supplementary motor area	R6	2	2	62	5.42
Superior temporal gyrus	L22	−56	−28	10	6.63
	R22	64	−24	6	5.58
Auditory cortex	L42	−54	−26	14	5.94
	R42	56	−36	10	7.27
**Correct rejection trials**					
Prefrontal cortex	R46	42	50	20	5.40
Premotor area	R6	52	8	44	5.69
Supplementary motor area	R6	2	6	60	6.64
Superior temporal gyrus	L22	−54	−20	4	9.78
	R22	54	−26	8	9.56
Auditory cortex	L42	−60	−34	12	5.98
	R42	64	−26	14	8.03
Supramarginal gyrus	R40	52	−38	50	7.78

*Activations of local maxima are significant at p < 0.001 (uncorrected; T > 3.47).*

*Coordinates (x, y, z) are in MNI stereotaxic space. BA, Brodmann area; L, left; R, right.*

**FIGURE 3:**
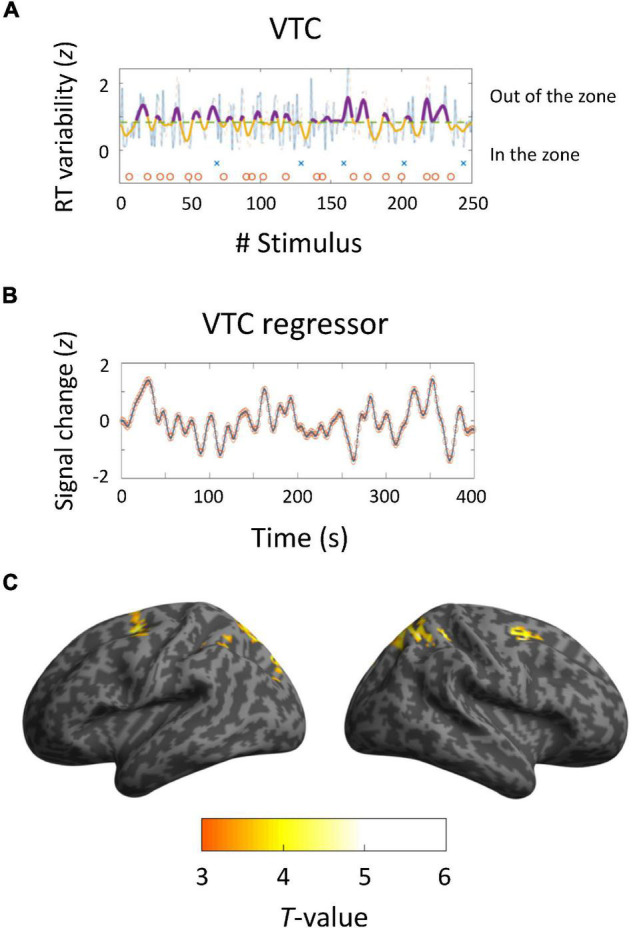
Brain activations derived from variance time courses (VTCs). **(A,B)** Behavioral and neuroimaging results of a representative participant. VTCs were computed using RTs for 250 trials. Dashed lines show median VTCs. The area below the line represents in-the-zone period, whereas the area above the line indicates out-of-the-zone period. Crosses and circles indicate false alarm and correct rejection trials, respectively. VTC regressors were convolved with a canonical hemodynamic response function and downsampled to 0.5 Hz. **(C)** Activated areas related to VTCs (*p* < 0.05, corrected at the cluster level; *k* = 71).

We computed the VTC for each run and produced regressors corresponding to the VTC convolved with an HRF (Figures 3A,B). The pattern of VTC-related activations differed from that of trial-based activations: the frontal eye field (BAs 8 and 6) and intraparietal sulcus (BA7) (Figure 3C). Local maxima were positioned in the coordinates (−26, 6, 56; *T* = 4.22) and (28, 6, 54; *T* = 5.06) for the SFG; (−12, −72, 58; *T* = 4.36) and (18, −66, 56; *T* = 5.02) for the intraparietal sulcus. There was no region negatively correlated with VTCs. Taken collectively, these results indicate that DAN regions are closely linked with modulation of sustained auditory attention.

### Brain Dynamics

**FIGURE 4:**
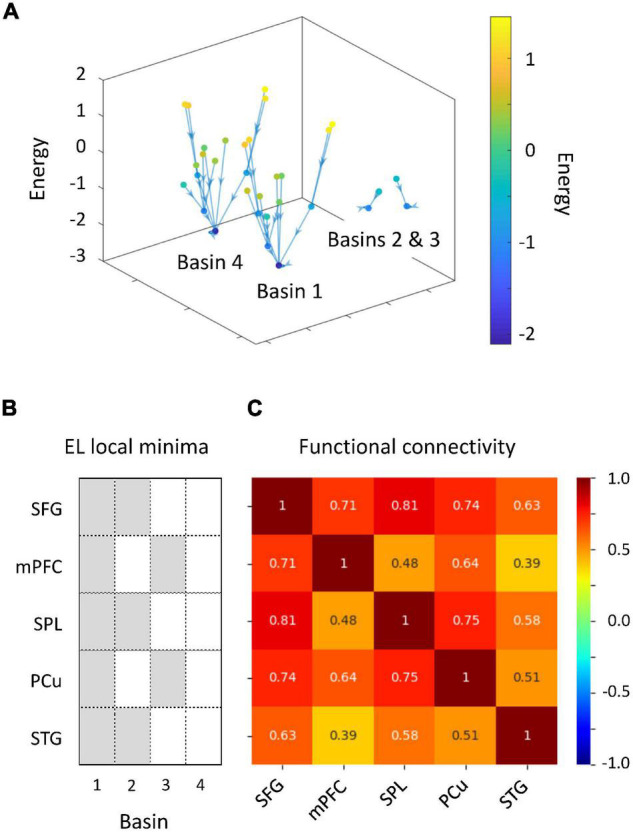
Results of energy landscape (EL) analysis. **(A)** Constructed EL. BOLD signals obtained from five regions of interests (ROIs) were binarized. Activity patterns were computed using the appearance probability of binarized states and categorized into four basins in EL. **(B)** ROI classification based on the disconnectivity graph. The EL transition rate was derived from activity patterns of active and inactive ROIs. **(C)** Comparison of EL local minima and functional connectivity.

We performed energy landscape analysis to characterize multivariate dynamics of the five ROIs. BOLD signals were binarized into −1 or 1, by which an activity pattern of these ROIs was represented by one of the possible 32 states. Based on binarized data, these activity patterns were clustered into “basins” of frequently visited states (local minima) (Figures 4A,B). We found four local minima. Specifically, two local minima (1 and 4) showed synchronized activity of ROIs (all active and inactive, respectively). Energy landscape analysis revealed two types of activity patterns in ROIs, whereas a general pattern of positive correlations between ROIs was found in terms of functional connectivity analysis (Figure 4C). These two activity patterns were classified into DAN and DMN regions. The activity pattern of the STG was associated with those of the DAN regions. Thus, we focused on dynamics between the two brain states (local minima 1 and 4).

**FIGURE 5:**
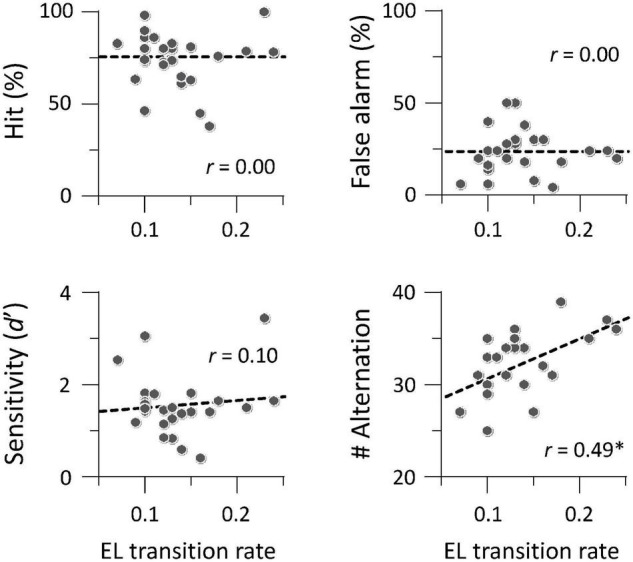
Brain-behavior relationship between transition rate and task performance (*N* = 26). The number of alternations between in- and out-of-the-zone periods was correlated with the transition rate between local minima 1 and 4 in energy landscape (EL) analysis. *p < 0.05 (Bonferroni correction for multiple comparisons).

**FIGURE 6:**
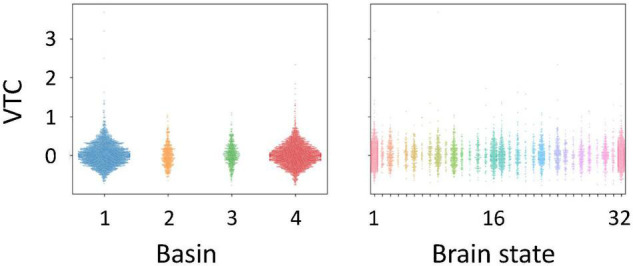
Variance time course (VTC) values identified for each basin and each brain state in the energy landscape. VTC values shown here were subtracted by the median VTC value of each run. Positive values correspond to out-of-the-zone periods.

We investigated whether auditory gradCPT performance was explained by the transition rate between the two brain states. We computed the transition rate by counting the number of direct transitions from basins 1 to 4 and from 4 to 1. The transition rate was positively correlated with the number of alternations between in- and out-of-the-zone periods: *r* = 0.49, *p* = 0.011 (Figure 5). When we calculated the transition rate between all basins, the correlation result was blurred: *r* = 0.18, *p* = 0.38. There was no correlation between the transition rate and other performance measures: hit rate, FA rate, and *d*′ (*r* < 0.10, *p* > 0.61). We did not find any systematic change in VTC values between basins and between brain states (Figure 6). In other words, there was no specific activity pattern that could be characterized by in- and out-of-the-zone periods. The results suggest that activations of the DAN and DMN regions, in addition to the STG, are involved in fluctuations in sustained auditory attention, although DAN and DMN activations are functionally differentiated.

## Discussion

We provide insights into dynamic brain mechanisms underlying sustained auditory attention. Event-related fMRI analyses demonstrated that frontal and temporal activations overlapped between successful and unsuccessful inhibition of predominant responses. RT variability of the auditory gradCPT was correlated with signal changes of DAN regions, including the SFG and SPL. The activation pattern of response inhibition differed from that of RT variability. Functional connectivity analysis confirmed that task-related activations were generally correlated. The energy landscape analysis produced major and minor brain states to further characterize the dynamics of these regions. Dynamics of DAN regions were synchronized with those of DMN regions in terms of major brain states, whereas dynamics of the DAN and DMN regions were functionally separable in terms of minor brain states. The number of alternations between in- and out-of-the-zone periods increased with an increase in the transition rate between the two major brain states. Taking VTC-related activity into account, we can imagine that neural mechanisms of sustained attention are probably dissociated from those of task performance itself. Therefore, we conclude that dynamic transitions between brain states are closely linked with auditory attentional fluctuations at the behavioral level.

Consistent with previous findings (Terashima et al., 2021), our results showed that alternations between successful and erratic periods ranged from 25 to 50 s. This suggests that attentional fluctuation is less susceptible to experimental environments and sensory disturbance. Intriguingly, lapses of attention during a Go/No-go task occur every 15–40 s (Vaurio et al., 2009), whereas temporal dynamics of perceptual switching have a shorter timescale, from several to 10 s (Pressnitzer and Hupé, 2006; Kondo et al., 2012). Spontaneous switching in multistable perception is based on formation and selection of perceptual objects, such as auditory streams (Gutschalk et al., 2005; Kondo and Kashino, 2009) and verbal forms (Sato et al., 2004; Kondo and Kashino, 2007). Thus, different biological rhythms are probably involved in perceptual organization and sustained attention, even though mental processes of administered tasks share the same auditory modality.

Auditory functions are supported by hierarchical processing in the brain. From a histological view, the auditory cortex comprises six subregions (Rivier and Clarke, 1997). We found that lateral and posterior areas of the auditory cortex were activated during changes in phonetic categories, that is, different genders of voices. Auditory-related activations extended to the lateral and posterior STG. Previous studies have demonstrated that the lateral area of the auditory cortex is associated with perceptual formation of speech envelope-modulated noises (Mummery et al., 1999) and triplet-tone sequences (Gutschalk et al., 2005; Kondo and Kashino, 2009). In addition, speech-related stimuli with phonetic cues, regardless of intelligibility, induce STG activity (Scott et al., 2000). The STG is also activated by phonetic reorganization of a stimulus word (Kondo and Kashino, 2007). Thus, the secondary auditory area is critical in change detection for both speech and non-speech stimuli. However, VTC-related activity was found in the SFG and SPL, but not in the auditory cortex and STG. It has been argued that selective attention to auditory perceptual organization is implemented in the intraparietal sulcus (Cusack, 2005). Volitional control to multistable perception is associated with the balance between neural inhibition and excitation in the intraparietal cortex (Kondo et al., 2018). These findings indicate that one of the DAN regions contributes to controlled attention in auditory domain. Thus, it is possible that neural correlates of auditory perceptual organization differ from those of auditory selective attention.

In general, achievement of task performance is required to maintain a constant level of attention. A classical study proposed that the attention system has three major functions: (a) orienting to events, (b) detecting signals for conscious processing, and (c) maintaining an alert state (Posner and Petersen, 1990). Several researchers have indicated that the ability to remain alert over time (sustained attention) is not necessarily the same as the ability to quickly change to an alert state (selective attention) (Petersen and Posner, 2012; Tang et al., 2015; Fortenbaugh et al., 2017). A resource-control model has been proposed to account for the nature of sustained attention (Thomson et al., 2015). This model postulates that a gradual reduction in task performance reflects a bias such that attentional resources are assigned to a default state. For the auditory gradCPT, it should be noted that RTs do not decrease monotonically, but vary with time. Trial-by-trial variability of task performance is frequently observed in humans even when a task is constant (Gilden, 2001). This may be the reason why VTC-related regions include DAN regions, rather than DMN regions.

Dynamic states of different attractors, including DAN, DMN, and STG regions, were linked to attentional fluctuation in the auditory gradCPT. Specifically, the transition rate between brain states was positively correlated with the number of alternations between in- and out-of-the-zone periods. This indicates that dynamics of brain states, rather than strength of brain activity, are important to predict moment-to-moment attentional levels. However, it is difficult to interpret what mental processes are directly reflected in such a brain state. We would like to emphasize that coarse-grained brain dynamics are important to explain fluctuations of sustained auditory attention.

Activity patterns of DAN and DMN regions were generally synchronized during the auditory gradCPT, but functionally separable. Dynamic states of brain regions did not differ between in- and out-of-the-zone periods. In contrast, it is well known that intrinsic BOLD signals of DAN regions are anti-correlated with those of DMN regions during rest (Fox et al., 2005). Previous studies using energy landscape analysis have revealed that dynamic transitions of brain states can predict individual differences in visual perception and personality traits. Transitions to frontal-area or visual-area-dominant states are associated with perceptual switching of a bistable structure-from-motion stimulus (Watanabe et al., 2014). Individuals with autism spectrum disorder have infrequent transitions between unstable intermediate states of brain networks (Watanabe and Rees, 2017). Thus, future studies should investigate how brain states change between task-positive and task-negative paradigms.

Brain activations time-locked to FA and CR trials were found in the right dorsolateral PFC, motor-related areas, and auditory-related areas. Meta-analysis studies have argued that the right inferior frontal cortex (Aron et al., 2004) and supplementary motor area (Simmonds et al., 2008) are responsible for response inhibition in Go/No-go tasks. From a methodological perspective, procedures of standard CPTs are similar to those of Go/No-go tasks that assess the ability to suppress unwanted actions or predominant responses (Ogg et al., 2008; Koizumi et al., 2018). In the dual-network model for top-down control (Dosenbach et al., 2007), the fronto-parietal network initiates and adjusts adaptive control on a trial-by-trial basis, whereas the cingulo-opercular network maintains a mental set throughout an entire task epoch. In addition, most studies on vigilance have investigated a linear decrease in brain activity (Olsen et al., 2013). It should be noted that the present study focused on moment-to-moment attentional fluctuations. Energy landscape analysis showed that temporal dynamics of DAN regions are synchronized with those of DMN regions, but that they are functionally separable from each other.

Various researchers have devoted their efforts to investigating transient attention, such as visual search (Treisman and Gelade, 1980), attentive object tracking (Pylyshyn and Storm, 1988), and attentional blink (Raymond et al., 1992). We focused on brain dynamics of sustained attention during auditory gradCPT. The frontal and temporal areas were responsible for intermittent response inhibition, whereas the DAN, DMN, and STG regions were involved in time-series RT variability. Energy landscape analysis revealed that dynamic transitions between brain states were closely linked to attentional fluctuation. Our findings can yield new insights into various types of research not only on perception and attention, but also on vigilance and mind wandering.

## Data Availability Statement

The raw data supporting the conclusions of this article will be made available by the authors, without undue reservation.

## Ethics Statement

The studies involving human participants were reviewed and approved by the Research Ethics and Safety Committees of Chukyo University and ATR-Promotions. The participants provided their written informed consent to participate in this study.

## Author Contributions

HMK, HT, KK, and JIK: conceptualization. HMK, HT, TE, and TK: data curation, formal analysis, methodology, resources, and writing—original draft. HMK, TE, KK, and JIK: funding acquisition. HMK and TK: investigation. HMK: project administration and supervision. HMK and TE: writing—review and editing. All authors contributed to the article and approved the submitted version.

## Conflict of Interest

HT was employed by NTT Corporation. TK was employed by ATR-Promotions. The remaining authors declare that the research was conducted in the absence of any commercial or financial relationships that could be construed as a potential conflict of interest.

## Publisher’s Note

All claims expressed in this article are solely those of the authors and do not necessarily represent those of their affiliated organizations, or those of the publisher, the editors and the reviewers. Any product that may be evaluated in this article, or claim that may be made by its manufacturer, is not guaranteed or endorsed by the publisher.

## Footnotes

1
http://www.fil.ion.ucl.ac.uk/spm
